# Mapping the landscape of brain stimulation research: A global scientometric review on cognitive impairment

**DOI:** 10.1002/ibra.12194

**Published:** 2025-03-22

**Authors:** Mani Abdul Karim

**Affiliations:** ^1^ Department of Psychology XIM University Bhubaneswar Odisha India

**Keywords:** bibliometrics, brain stimulation, CiteSpace, cognitive impairment, scientometrics, transcranial magnetic stimulation

## Abstract

Several noninvasive brain stimulation techniques have gained significant attention in neurocognitive science and clinical research due to their potential efficacy in addressing neurological, psychiatric, and cognitive impairments. This study explores global trends and research hotspots in brain stimulation research for cognitive impairment and related disorders. Using a data set from 1989 to 2024 sourced from the Web of Science Core Collection, 4156 records were analyzed through bibliometric methods, including publication trends, country or region, and institutional analysis, and document co‐citation analysis (DCA). Results revealed a steady increase in research, with a significant increase in publications during the period from 2019 to 2023. The USA led in citation counts (1117), centrality (0.37), while China topped the burst value (72.31). The University of London led in citation counts (235), whereas Capital Medical University topped the sigma value (1.77). Transcranial magnetic stimulation (TMS) and repetitive TMS (rTMS) dominated the top positions in DCA analysis. Emerging trends were identified through burst keywords, including “transcranial Doppler,” “subthalamic nucleus stimulation,” “cerebral blood flow,” “vascular dementia,” and “cardiopulmonary bypass.” These emerging research hotspots underscore the growing focus on vascular aspects of cognitive impairment and advanced brain stimulation methods. Additionally, newer noninvasive techniques like fast gamma magnetic stimulation, paired‐associative stimulation with TMS (PAS‐TMS), and theta‐burst stimulation are identified as promising avenues for future research, offering significant potential for therapeutic advancements. This study provides a comprehensive overview of the global landscape, trends, and future directions in brain stimulation research for cognitive impairment.

## Graphical abstract

This study maps global trends in brain stimulation research for cognitive impairment using bibliometric analysis of 4156 records (1989–2024, WoSCC). Findings highlight the dominance of transcranial magnetic stimulation (TMS) and repetitive TMS (rTMS), emerging vascular‐focused research, and promising techniques like fast gamma magnetic stimulation and paired‐associative stimulation with TMS. The analysis provides insights into key contributors, research hotspots, and future directions in the field.

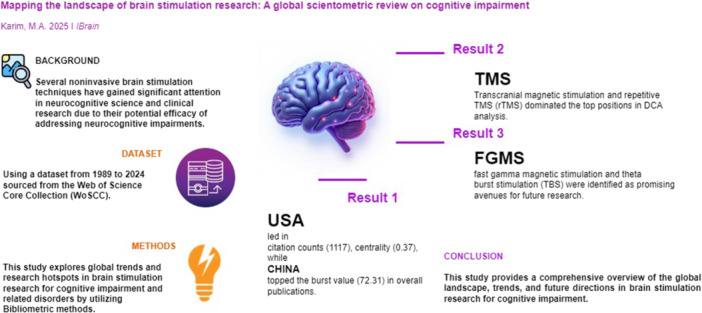

## 1 INTRODUCTION

Cognitive impairment constitutes a fundamental attribute shared by a diverse range of neuropsychiatric disorders, characterized by deficits in memory, executive functions, attention, language, and problem‐solving abilities.^1^ This decline goes beyond normal aging and can impede an individual's daily activities, particularly when the impairment attains a moderate or severe level. It can appear in various forms, including mild cognitive impairment (MCI), also known as mild neurocognitive disorders,^2^ and more severe conditions like major neurocognitive disorders (NCDs), Alzheimer's Disease (AD),^3^, ^4^, ^5^ vascular dementia,^6^ Lewy body dementia (LBD), frontotemporal lobar degeneration (frontotemporal dementia),^7^, ^8^ traumatic brain injury,^9^, ^10^, ^11^ and Parkinson's disease (PD).^12^, ^13^, ^14^

Among these, MCI represents an early stage of cognitive decline, affecting an estimated 15%–40% of adults aged 65 and older.^15^, ^16^, ^17^, ^18^, ^19^ AD, a prominent cause of dementia, impacts approximately 5% of individuals aged 65 and older, escalating to almost 32% among those aged 85 and older.^20^, ^21^ Parkinson's disease dementia, and LBD also contribute to the overall prevalence of cognitive impairment.^22^, ^23^, ^24^, ^25^, ^26^ The early diagnosis and the implementation of interventions, particularly noninvasive brain stimulation techniques, play a crucial role in mitigating cognitive impairment and enhancing the quality of life for older individuals affected by these conditions.

Several noninvasive brain stimulation techniques and its recent developments have gained increasing attention in cognitive neuroscience and clinical research to treat cognitive imapirment.^4^, ^27^, ^28^ Brain stimulation techniques such as transcranial magnetic stimulation (TMS), repetitive TMS (rTMS), transcranial direct current stimulation (tDCS), transcranial alternating current stimulation (tACS), functional near‐infrared spectroscopy (fNIRS), electroencephalography (EEG), and theta burst stimulation (TBS), are involved in the application of external stimuli to modulate neural activity without the need for surgery or implantation. TMS, for instance, uses magnetic fields to induce electric currents in specific brain regions and subsequently alters neuronal activity. This method of stimulation has been shown to reduce neuropsychiatric symptoms, improve cognition, motor learning, attention, associative memory, being a potential method of treatment for cognitive symptoms associated with dementia.^29^, ^30^, ^31^ Similarly, tDCS involves delivering a low electrical current through electrodes on the scalp to modulate neuronal activity which try to improve the efficacy in cognitive enhancement and various neurological disorders.^32^, ^33^, ^34^ The another techniques such as fNIRS and EEG, are used to explore brain function during various cognitive tasks and activities implement while act as a real‐time feedback on brain activity to enable individuals to self‐regulate their neural patterns.^35^, ^36^, ^37^, ^38^, ^39^, ^40^, ^41^

Despite the growing interest in the noninvasive brain stimulation techniques, there is a need for deeper insight into the global trends and emerging hotspots in brain stimulation research. The uniqueness of this study lies in its utilization of bibliometric analysis, offering a systematic and quantitative approach to map and analyze the extensive literature. By employing advanced software (CiteSpace) and various bibliometric methods such as co‐citation pattern analysis, this study provides a nuanced perspective on publication trends, institutional contributions, author impact, and emerging keywords, offering valuable insights into the evolving landscape of brain stimulation research. Based on the research gap, the study aims to:

1. Identify primary research themes and global recent trends in noninvasive brain stimulation for cognitive impairment.
2. Analyze the evolution of brain stimulation research over the past three decades.
3. Assess key geographic regions contributing to research on brain stimulation for cognitive impairment.
4. Identify influential researchers and institutions contributing to the field of brain stimulation research.
5. Understand the interdisciplinary connections based on dual map overlay analysis.
6. Identify the emerging research clusters and topics within brain stimulation research and co‐citation patterns.
7. Provide a comprehensive overview of scholarly discourse on evolving landscape of brain stimulation research.

## 2 METHODS AND MATERIALS

Several databases, including Web of Science (WoS), Scopus, and Google Scholar, offer social sciences citation indexing services. However, in this study, WoS was selected for its quality and the advanced search features.^42^ The WoS Core Collection (WoSCC)‐Citation database provides unique citation count features, facilitating article importance assessment. Therefore, bibliometric researchers frequently use WoSCC for scientific publication analysis. The study utilizes a recent version of CiteSpace, which is more apted to visualize the WoSCC data, making the neutral and informative choice for this bibliometric research.

### 2.1 Data sources and searching strategies

The data set was gathered from the WoSCC database, comprising scientific publications indexed by the social sciences citation index (SSCI), science citation index expanded (SCI‐E), and arts & humanities citation index. This collection is esteemed as the definitive source of scientific publications and high‐quality articles across various research domains. The ensuing search strategies were utilized to compile the data set.

#### 2.1.1 Search strategy 1 (SET‐1)

TS = (“mild cognitive impairment”) OR TS = (“cognitive impairment”) OR TS = (“cognitive dysfunction”) OR TS = (“benign senescent forgetfulness”) OR TS = (“age‐associated memory impairment”) OR TS = (“late‐life forgetfulness”) OR TS = (“age‐associated cognitive decline”) OR TS = (dementia) OR TS = (“Alzheimer's disease”). A total of 394,052 data were retrieved in this search strategy (Session: August 5, 2024).

#### 2.1.2 Search strategy 2 (SET‐2)

TS = (“cognitive stimulation”) OR TS = (“transcranial magnetic stimulation”) AND TS = (stimulation) OR TS = (transcranial*) OR TS = (“brain stimulation”) OR TS = (tDCS*) OR TS = (“transcranial direct current stimulation”) OR TS = (“transcranial random noise stimulation”) OR TS = (tRNS*) OR TS = (“transcranial alternating current stimulation”) OR TS = (tACS*) OR TS = (“brain stimulation therapy”) OR TS = (“deep brain stimulation”). A total of 91,281 data were retrieved in this search strategy (Session: August 5, 2024).

#### 2.1.3 Search strategy 3 (SET‐3): SET‐1 AND SET‐2

**Figure 1:**
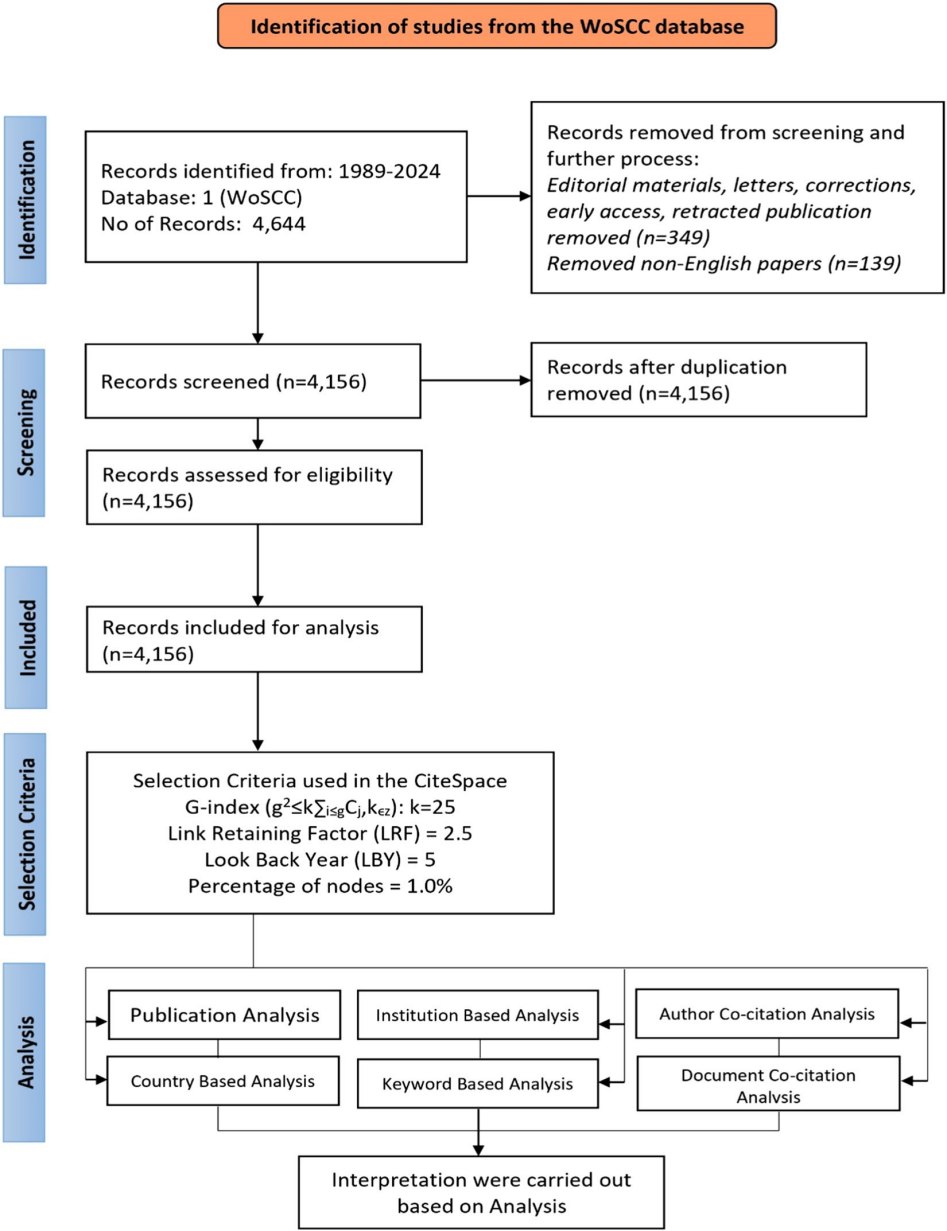
PRISMA flow diagram for scientometric review. PRISMA, Preferred Reporting Items for Systematic reviews and Meta‐Analyses; WoSCC, Web of Science Core Collection. [Color figure can be viewed at wileyonlinelibrary.com]

A total of 4644 data were collected by merging Strategy 1 and Strategy 2, spanning from 1989 to 2024 (retrieved on August 5, 2024). Subsequently, the collected data underwent refinement, which involved including only articles, review articles, proceeding papers, and book chapters, while excluding editorial material, letters, and corrections. Additionally, all non‐English language entries were excluded. Consequently, 4156 datasets were finally selected for bibliographic analysis. The consolidated data included article titles, author names, abstracts, keywords, and cited references. The preferred reporting items for systematic reviews and meta‐analyses (PRISMA) flow diagram illustrating the search strategy, sorting process of relevant articles, and selection criteria applied in CiteSpace is depicted in Figure 1.

### 2.2 Bibliometric method and its measurement

Bibliometric analysis is a quantitative method used to evaluate and analyze academic publishing through citation analysis. It involves examining patterns and trends within publications, citations, authors, journals, and other bibliographic elements to gain insights into the structure and dynamics of scholarly communication.^43^, ^44^ The most prevalent units include journals, authors, cited references, documents, institutions, and countries.^45^

Microsoft Excel 2013 and CiteSpace 6.3.R3 (64‐bit), an advanced software version, were utilized for mapping and analyzing recent trends and patterns in this study.^43^, ^46^, ^47^, ^48^, ^49^ The bibliometric analyses encompass document co‐citation analysis (DCA), author co‐citation analysis (ACA), network of country distribution, institutions distribution, and co‐occurrence of the keyword analysis. Both DCA and ACA in CiteSpace were employed to construct a matrix of cited links, which were then grouped into clusters representing intellectual bases.^43^, ^47^, ^49^ These clusters provide insights into current trends and intellectual milestones. Additionally, bibliometric analysis serves as a convenient tool for researchers to stay informed about the latest trends in the field.

CiteSpace considers various structural and temporal measures for analyzing document or author co‐cited references and generating clusters. In this study, four measures were adopted to assess cluster homogeneity (Silhouette value), sudden changes (burst measurement), centrality of the network (betweenness centrality), and scientific novelty (Sigma measurement). Silhouette values range from −1 to +1, with a value of 1 indicating perfect separation between clusters.^43^, ^46^, ^50^ The burst detection algorithm quantifies the frequency of sudden increases in references, while the sigma value measures scientific novelty.^46^, ^51^ Centrality estimates the importance of a node or edge for network connectivity or information flow.^46^, ^50^, ^52^

## 3 RESULTS

### 3.1 Annual trend

**Figure 2:**
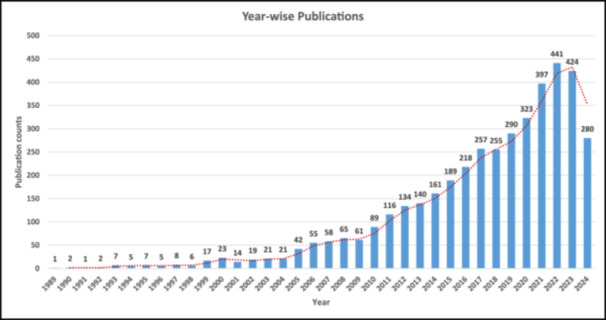
The number of annual publications on cognitive stimulation studies for cognitive impairment and related disorders from 1989 to 2024. *Note:* The trendline shows the two period moving average. [Color figure can be viewed at wileyonlinelibrary.com]

After screening, this study finally retrieved a total of 4156 literatures in the WoSCC database from 1989 to 2024 for bibliometric analysis. It is important to note that the publication data for 2024 is incomplete, as the literature search was conducted before the year's conclusion (August 5, 2024). Therefore, the 2024 publication count of 280 articles does not reflect the full year's output and is excluded from the discussion of annual trend. Notably, there has been a significant increase in publications observed during the period from 2019 to 2023, with a total of 1875 articles published, accounting for 45.12% of the total articles published within the broader timeframe of 1989 to 2024 (Figure 2). This notable rise in the number of published documents could be attributed to the increased attention and recognition received by numerous articles within the scientific community. Particularly, after the COVID‐19 pandemic period, with the year 2022 witnessing the highest number of publications since 1989, totaling 441 articles.

### 3.2 Most influential countries or regions

The number of articles published by each country or region during the span of 1989 to 2024 possesses describing the impact of the most productive geographical regions or countries in this study domain. In country‐based analysis, the modularity *Q*‐value of 0.8726, weighted mean Silhouette *S*‐value of 0.9472, and the harmonic mean (*Q*, *S*) of 0.9084, suggest that the network structure is highly significant and network homogeneity is relatively high (Supporting Information S1: Figure 1).

Through this analysis, several important bibliometric values were considered to identify the outstanding countries in terms of betweenness centrality, novel contribution (sigma), and high citation counts (Supporting Information S1: Table 1). The top 10 countries or regions based on citation counts were led by the USA (1117), followed by China (626), Italy (598), England (524), Germany (377), Canada (318), Spain (253), Australia (233), France (199), and Netherlands (146). The country or region with the highest burst value was China (72.31), followed by Wales (9.08), Germany (7.78), Austria (6.56), Scotland (4.25), USA (4.15), Japan (3.94), France (3.87), Belgium (3.33), and Hungary (3.19). Additionally, the USA ranked first in centrality (0.37), followed by Egypt (0.20), France (0.16), England (0.13), and Germany (0.10). Finally, the USA also obtained the highest Sigma value (3.68), followed by Germany (2.16), China (1.79), France (1.78), and England (1.34). In summary, this geographical region or country‐based scientometric analysis identifies the USA as a dominant force, leading in citation counts, centrality, and sigma value. Other notable contributors include Wales, Germany, Italy, and China, reflecting diverse global contributions to the cognitive stimulation‐based studies.

### 3.3 Most influential institutions

**Figure 3:**
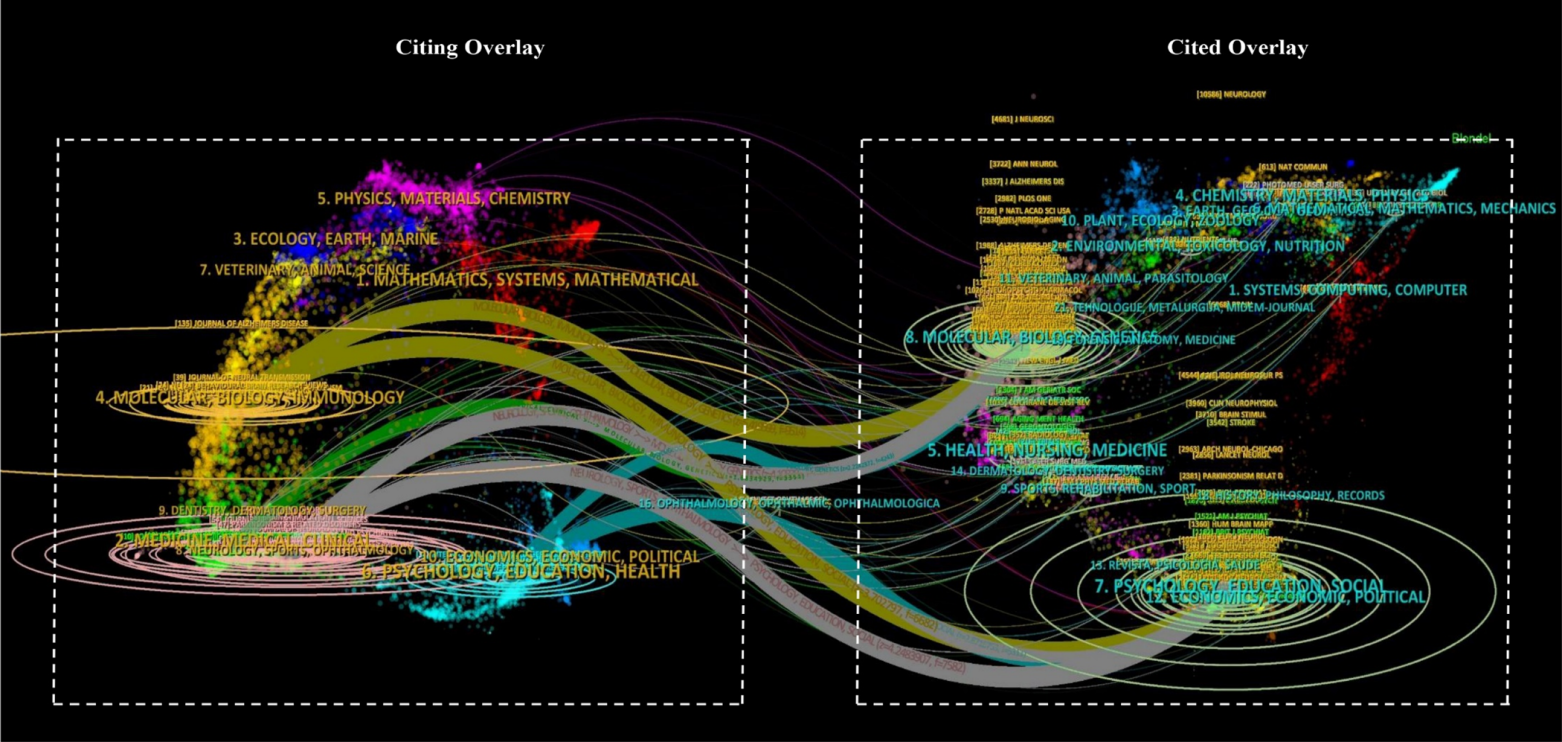
The dual‐map overlay constructed by using data set derived from web of science core collection (WoSCC) regards to cognitive stimulation studies related to cognitive impairment and related disorders between 1989 and 2024. *Note:* Each arrow represents strong path between the two disciplines. Left side is considered as the citing overlay, whereas right side is cited overlay. [Color figure can be viewed at wileyonlinelibrary.com]

Based on Institution‐wise analysis (Supporting Information S1: Figure 2 and Table 2), the five top‐ranked institutions by citation counts were University of London (235), Harvard University (183), University College London (180), University of Toronto (171), and Harvard Medical School (139). Assessing centrality, the Assistance Publique Hopitaux Paris (APHP; 0.10) emerges as the foremost institution, followed by Capital Medical University (0.09), INSERM (0.06), University of Pennsylvania (0.06), and Johns Hopkins University (0.06). Based on the Sigma value, the first institution during this period was Capital Medical University (1.77), followed by APHP (1.49), University of Manchester (1.36), University of Pennsylvania (1.22), and University of Barcelona (1.22). Based on burst value (Supporting Information S1: Figure 3), University of Manchester secured the highest burst value (12.36) in this domain, followed by Helmholtz Association (8.12), Bangor University (7.78), CIBERNED (7.60), and University of Verona (6.92). In summary, the University of London and the University of Manchester, both in the United Kingdom, stand out for their citation counts and sudden increase in the citation frequency (burst value), whereas the APHP led in centrality value, and Capital Medical University in China leads in sigma value.

### 3.4 Most eminent authors

The study delineates the most influential authors in brain stimulation research for cognitive impairment and related disorders, as evidenced by their publication records, citation counts, centrality, and burst value (Supporting Information S1: Table 3). A total of 18,345 authors contributed to 4156 publications in this study domain. Leading in terms of publication records is Author *Spector, A* (60), whose primary focus revolves around cognitive stimulation therapy program (CST).^53^, ^54^ Following closely is *Orrell, M*, (48), noted for articles on CST with dementia‐affected older adults and the development of individualized CST.^55^, ^56^, ^57^ Securing the third position is Author *Lozano, AM*, (46), recognized for contributions to research on deep brain stimulation (DBS). The author discuss the underlying mechanisms and the prospects of DBS in altering the course of AD.^13^, ^58^, ^59^, ^60^, ^61^ Author *Pascual‐leone*, A, (45), collaborated with other researchers and conducted various studies to critically examine the potential effect of various brain stimulation techniques to treat neuropsychiatric conditions, including major depression, AD, schizophrenia, autism, and attention‐deficit hyperactivity disorder (ADHD).^28^, ^62^, ^63^ Author Koch^64^, ^65^, ^66^ explored the use of TMS, TBS, and rTMS to improve synaptic dysfunction, enhance episodic memory, and treat memory impairments in patients with early‐stage AD.

**Figure 4:**
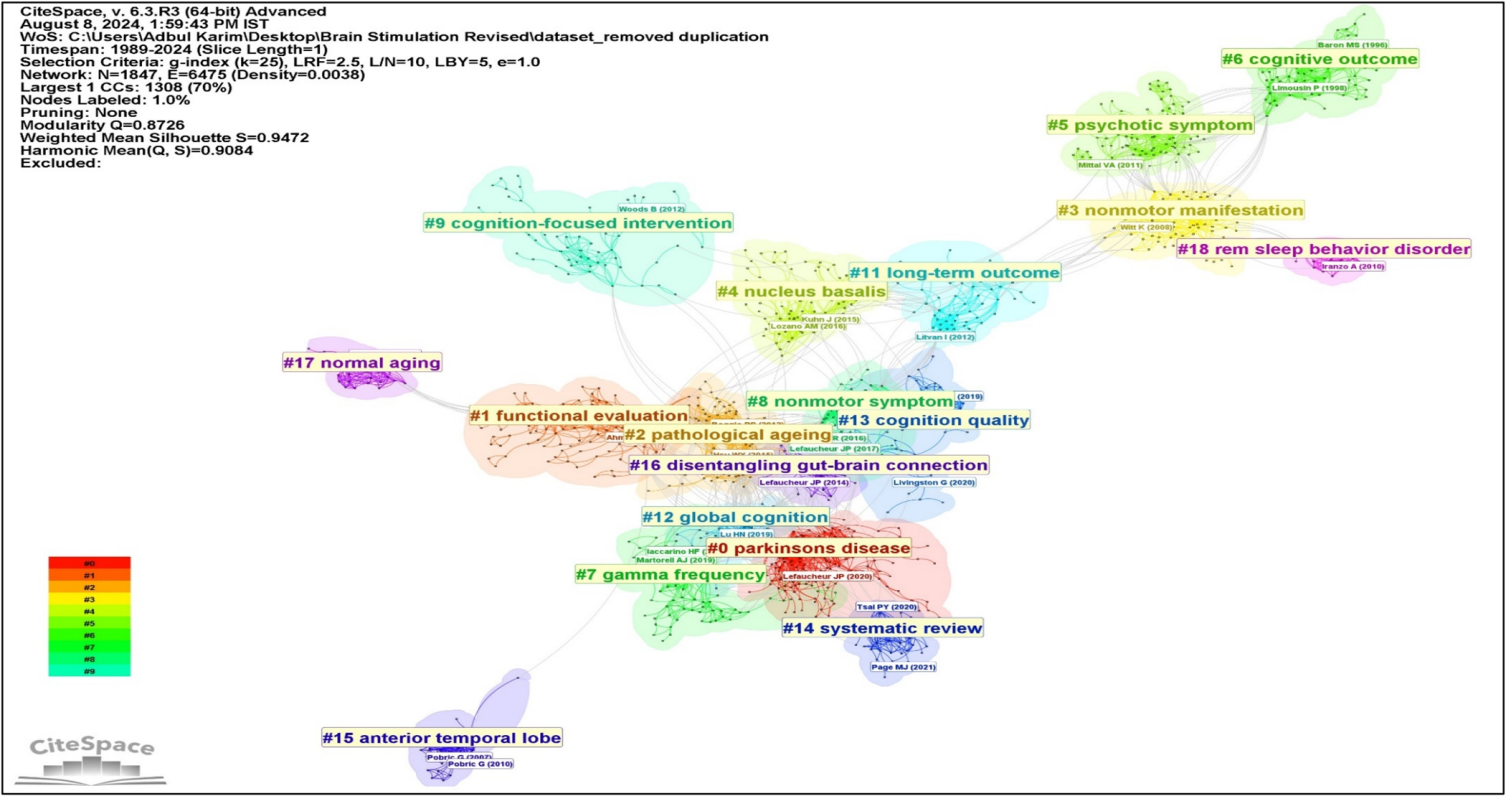
The landscape mapping view of clusters based on document co‐citation analysis (DCA) on brain stimulation studies for cognitive impairment and related disorders, generated by top 50 per slice between 1989 and 2024 (LRF = 2.5, LBY = 5, e = 1.0). Each color represents different cluster. LBY, look back year; LRF, link retaining factor. [Color figure can be viewed at wileyonlinelibrary.com]

Furthermore, an analysis was conducted based on citation count, centrality, and burst value. *Folstein, MF* (557), *Petersen, RC* (315), *and Nitche, MA* (312) are among the most cited for their contributions to cognitive assessment and noninvasive stimulation techniques (Supporting Information S1: Table 3 and Figure 4). In terms of centrality, *Aaslid, R* (0.15), *Dubois, B* (0.09), and *Barker, AT* (0.08) are noted for their foundational work in brain imaging, diagnostics, and stimulation methods. Burst analysis underscores recent breakthroughs by *Chou, YH* (32.80), *McKhann, G* (28.09), and others in brain stimulation and Alzheimer's research. Together, these authors have driven significant progress in both theoretical frameworks and practical treatments for cognitive impairments (Supporting Information S1: Table 3).

### 3.5 Keywords co‐occurrence

**Figure 5:**
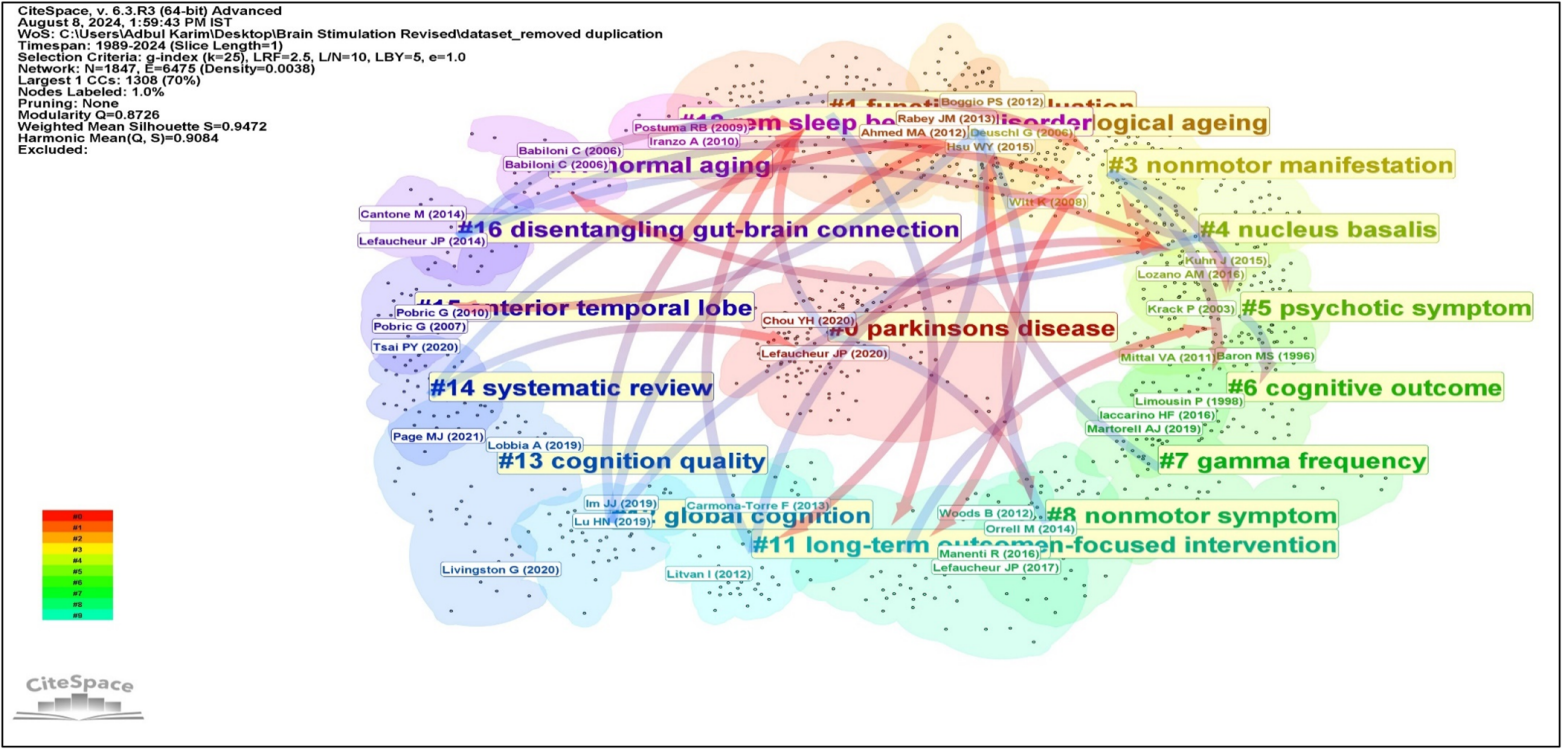
The landscape mapping centered circular view of cluster dependencies based on document co‐citation analysis (DCA), generated by top 50 per slice between 1989 and 2024 (LRF = 2.5, LBY = 5, e = 1.0) in studies related to cognitive stimulation. *Note:* The arrow marks show the cluster dependencies. LRF, link retaining factor; LBY, look back year. [Color figure can be viewed at wileyonlinelibrary.com]

Identifying influential keywords within a specific research domain is crucial for understanding emerging trend‐setting terms. Citation counts reflect the direct measure of how frequently a particular keyword is utilized by researchers, while betweenness centrality measures the position that a particular keyword occupies within the network structure (Supporting Information S1: Figure 5 and Table 4). Finally, the burst value or burstness algorithm identifies the trend of themes in academic publishing over time within a domain. The top ten most common keywords based on citation counts were “Alzheimer's disease (1398),” “dementia (833),” “transcranial magnetic stimulation (819),” “deep brain stimulation (789),” and “Parkinson's disease (678).” Keywords exhibiting high centrality were “dementia (0.11),” “cerebral blood flow (0.10),” “Alzheimer's disease (0.09),” “brain (0.09),” and “transcranial doppler (0.06).” According to the Burstness view of CiteSpace, the strongest citation bursts were observed in “transcranial doppler (26.51),” “subthalamic nucleus stimulation (14.99),” “cerebral blood flow (14.11),” “vascular dementia (14.00),” and “cardiopulmonary bypass (13.45)” during the period from 1989 to 2024 within the cognitive stimulation domain.

### 3.6 Dual‐map overlay analysis

The dual‐map overlay analysis is a method used in bibliometric analysis to visually depict the connections between two different sets of bibliographic data (citing overlay and cited overlay). In this analysis, two maps are overlaid on each other to reveal connections and intersections between different subject areas and disciplines. This approach proves particularly valuable in identifying robust connections within the interdisciplinary aspects of a specific research domain. Furthermore, it can help researchers in pinpointing emerging interdisciplinary research topics, tracking the diffusion of knowledge across disciplines, and guiding strategic decisions related to research funding, collaboration, and policy‐making.

As depicted in Figure 3, publications related to cognitive stimulation were published across four major disciplines. Notable disciplines on the citing overlay side included molecular, biology, immunology (MBI), medicine, medical, clinical (MMC), neurology, sports, ophthalmology (NSO), and psychology, education, health (PEH). Conversely, on the cited overlay side, two major disciplines stood out such as molecular, biology, genetics (MBG) and psychology, education, social (PES). Among the four major disciplines in the citing overlay, three clusters of disciplines such as MBI, NSO, and PEH demonstrated strong interconnections with both major disciplines in the cited overlay (MBG and PES). However, the green‐colored path arrow was linked only to MBG, but not PES. This indicates that studies based on brain stimulation are largely interdisciplinary in nature. Nonetheless, there exists a notable research gap in connecting MMC and PES, indicating a potential area for focused attention within the interdisciplinary network.

### 3.7 Most‐cited and influenced articles

Using a single‐year time slice, data set from 1989 to 2024, comprising 4156 published records, was visualized for DCA analysis. For each slice, the top 50 most cited articles were selected, resulting in a data set featuring 1847 nodes and 6475 links for DCA analysis. The visual representation of the overall network is illustrated in Figure 4. The overall mean Silhouette value for the network was exceptionally high at 0.9472, indicating a high degree of homogeneity of overall clusters formation.

**Figure 6:**
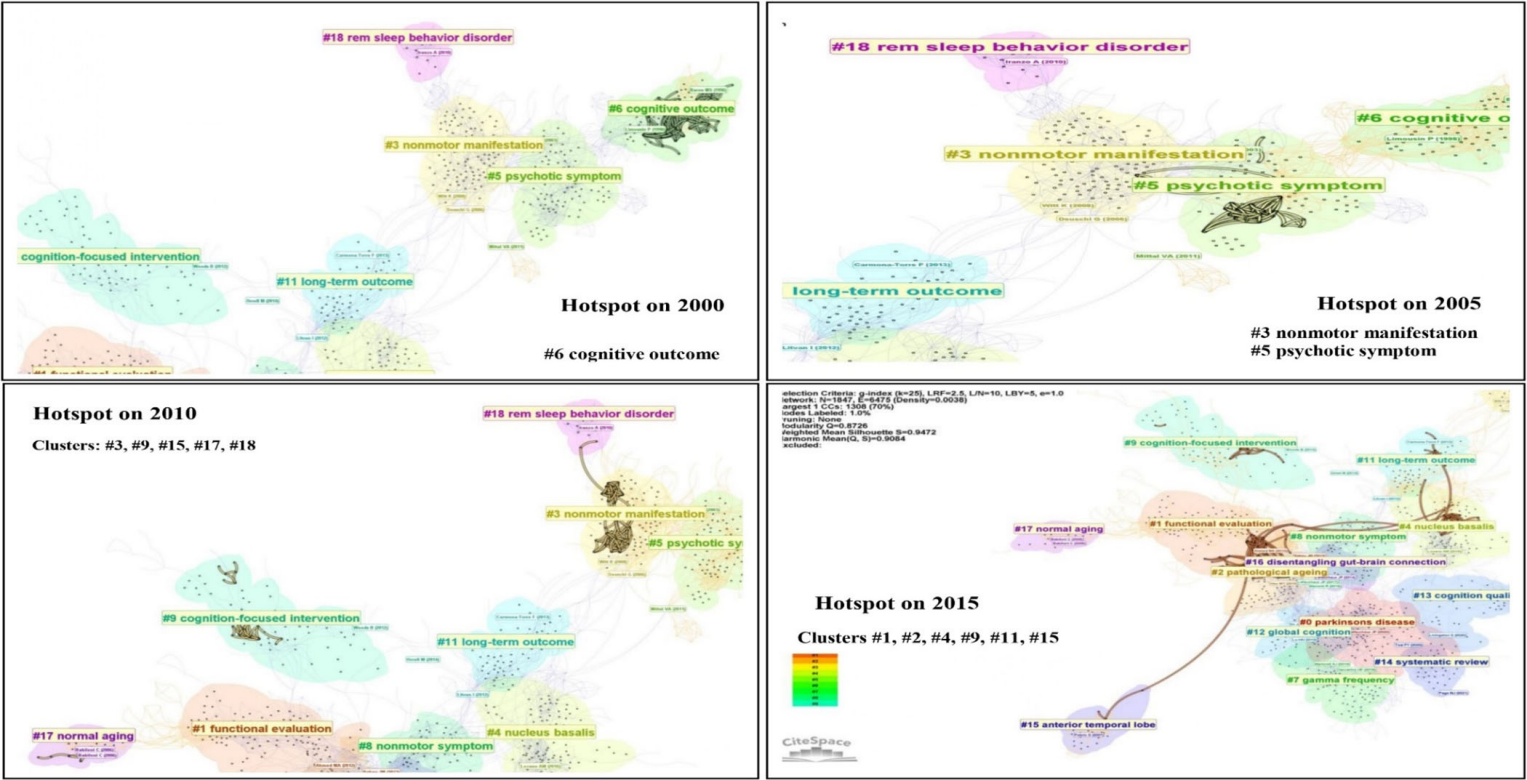
The multiple landscape mapping view of clusters across different periods from 2000 to 2015 based on document co‐citation analysis (DCA) on cognitive stimulation studies, generated by top 50 per slice between 1989 and 2024 (LRF = 3.0, LBY = 5, e = 1.0). The thick connection between clusters shows that studies were carried out predominantly (hotspot) on that particular cluster in different years. *Note: #1‐functional evaluation, #2‐pathological ageing, #3‐nonmotor manifestation, #4‐nucleus basalis, #5‐psychotic symptom, #6‐cognitive outcome, #9‐cognition‐focused intervention, #11‐long‐term outcome, #15‐anterior temporal lobe, #17‐normal aging, #18‐rem sleep behavior disorder*. LRF, link retaining factor; LBY, look back year. [Color figure can be viewed at wileyonlinelibrary.com]

**Figure 7:**
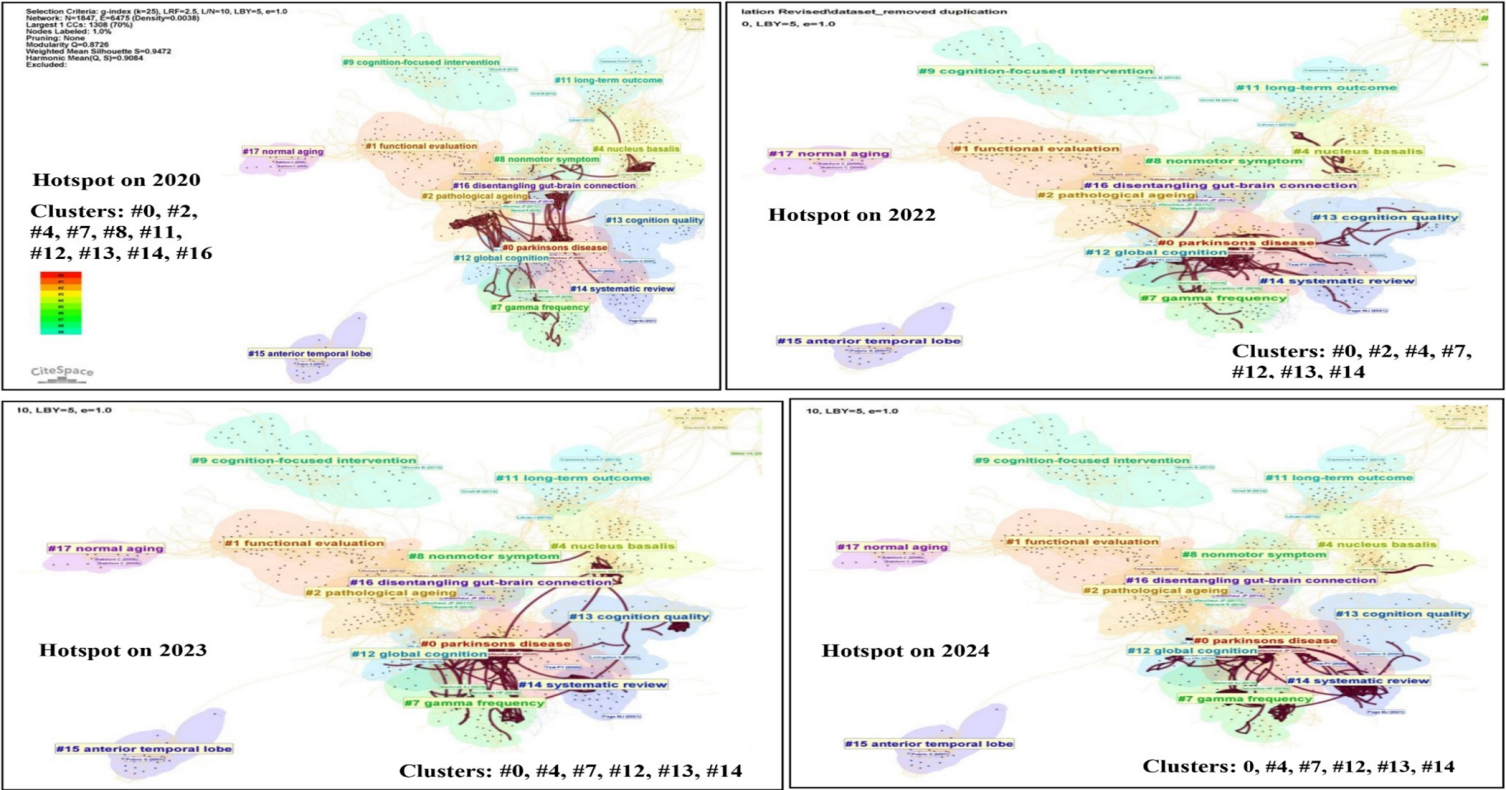
The multiple landscape mapping view of clusters across different periods from 2020 to 2024 based on document co‐citation analysis (DCA) on cognitive stimulation studies, generated by top 50 per slice between 1989 and 2024 (LRF = 3.0, LBY = 5, e = 1.0). The thick connection between clusters shows that studies were carried out predominantly (hotspot) on that particular cluster in different years. *Note: #0‐parkinsons disease, #2‐pathological ageing, #4‐nucleus basalis, #7‐gama frequency, #8‐nonmotor symptoms, #11‐long‐term outcome, #12‐global cognition, #13‐cognition quality, #14‐systematic review, #16‐disentangling gut‐brain connection*. LRF, link retaining factor; LBY, look back year. [Color figure can be viewed at wileyonlinelibrary.com]

In this DCA analysis, three significant approaches were conducted: clustering based on the most‐cited influential articles in this domain (Figure 4 and Table 1), cluster dependencies (Figure 5), and cluster transformation over time (Figures 6, 7). Cluster exploration was conducted using CiteSpace software with the time span of 1989 to 2024 (Slice length = 1), employing specific selection criteria including a g‐index (*k* = 25), link retaining factor (LRF = 2.5), link‐to‐node ratio (L/N ratio) of 10, and a look back year (LBY = 5). After DCA analysis, the top five clusters were listed in Table 1, based on citation count, burst value, betweenness centrality, and sigma value. CiteSpace software automatically labeled cluster names using log‐likelihood rate (LLR), with the second retaining theme name applied if duplicate cluster names were present.

**Table 1:** The top‐5 clusters and overall DCA analysis on finding research trends in brain stimulation studies between 1989 and 2024, listed with citation counts (CC), centrality (σ), and Sigma value.

Cluster ID	CS	SMV	Mean (Year)		CC	DOI of cited reference		B	DOI of Cited reference		σ	DOI of Cited reference		Σ	DOI of Cited reference		
Parkinson's disease (0)	162	0.902	2018	**Top‐5 publications based on citation count**	106	10.1016/j. neurobiolaging.2019.08.020	**Top‐5 publications based on Burst value**	30.04	10.1016/j. neurobiolaging.2019.08.020	**Top‐5 publications based on Centrality value**	0.07	‐	**Top‐5 publications based on Sigma value**	2.66	‐		
					102	10.1016/j. clinph.2019.11.002		29.49	10.1016/j. clinph.2015.02.001		0.05	10.1016/j. jalz.2018.02.018		1.91	10.1016/j. jalz.2018.02.018		
					96	10.1016/j. neuroimage.2017.12.048		27.39	10.1016/j. clinph.2019.11.002		0.03	10.1002/ana.24695		1.76	10.1016/j. clinph.2019.11.002		
					70	10.1016/j. clinph.2015.02.001		23.93	10.1016/j. neuroimage.2017.12.048		0.02	10.1016/j. clinph.2015.02.001		1.66	10.1016/j. neuroimage.2017.12.048		
					55	10.1016/j. jalz.2018.02.018		18.51	10.1016/j. brs.2021.01.012		0.02	10.1016/j. clinph.2019.11.002		1.62	10.1016/j. clinph.2015.02.001		
Functional evaluation (1)	121	0.936	2008		43	10.1007/s00415‐011‐6128‐4		21.03	10.1007/s00415‐011‐6128‐4		0.14	10.1007/s00702‐012‐0902‐z		13.46	10.1007/s00702‐012‐0902‐z
					42	10.1007/s00702‐012‐0902‐z		19.83	10.1007/s00702‐012‐0902‐z		0.10	10.1136/jnnp.2009.197848		5.84	10.1136/jnnp.2009.197848
					36	10.1136/jnnp.2009.197848		18.49	10.1136/jnnp.2009.197848		0.09	10.1007/s12017‐009‐8109‐7		1.76	10.3233/JAD‐2012‐120532
					32	10.1007/s00702‐010‐0578‐1		17.37	10.1007/s00702‐010‐0578‐1		0.07	10.1016/j. jns.2014.01.037		1.73	10.1038/npp.2009.60
					29	10.1016/j. clinph.2009.08.016		16.21	10.1016/j. clinph.2009.08.016		0.06	10.3233/JAD‐2012‐120532		1.54	10.1016/j. jns.2014.01.037
Pathological ageing (2)	113	0.915	2013		62	10.1016/j. neurobiolaging.2015.04.016		26.09	10.1016/j. neurobiolaging.2015.04.016		0.09	10.3389/fnagi.2014.00038		2.29	10.3389/fnagi.2014.00038	
					40	10.1186/s13195‐014‐0074‐1		20.90	10.1016/j. brs.2011.06.006		0.04	10.1016/j. neuroimage.2013.05.098		1.86	10.1016/j. neurobiolaging.2015.04.016	
					40	10.1016/j. brs.2011.06.006		18.11	10.1186/s13195‐014‐0074‐1		0.03	10.1186/s13195‐016‐0180‐3		1.59	10.1186/s13195‐016‐0180‐3	
					34	10.1186/s13195‐016‐0180‐3		14.15	10.1016/j. jalz.2014.07.159		0.03	10.1016/j. arr.2016.05.006		1.50	10.1016/j. neuroimage.2013.05.098	
					31	10.1016/j. jalz.2014.07.159		13.86	10.1186/s13195‐016‐0180‐3		0.03	10.3389/fnhum.2012.00046		1.33	10.1016/j. brs.2011.06.006	
Nonmotor manifestation (3)	108	0.954	2005		23	10.1016/S1474‐4422(08)70114‐5		13.62	10.1056/NEJMoa060281		0.15	10.1056/NEJMoa060281		6.91	10.1056/NEJMoa060281
					23	10.1056/NEJMoa060281		13.31	10.1016/S1474‐4422(08)70114‐5		0.05	10.1002/mds.20963		1.56	10.1136/jnnp.2002.009803
					21	10.1136/jnnp.2002.009803		13.01	10.1136/jnnp.2002.009803		0.04	10.1056/NEJMoa041470		1.44	10.1056/NEJMoa041470
					18	10.1002/mds.21507		10.36	10.1002/mds.21507		0.04	10.1002/mds.21478		1.42	10.1002/mds.20963
					16	10.1016/S1474‐4422(06)70475‐6		09.68	10.1016/S1474‐4422(06)70475‐6		0.03	10.1136/jnnp.2002.009803		1.22	10.1002/mds.21507
Nucleus basalis (4)	102	0.962	2015		73	10.3233/JAD‐160017		26.40	10.3233/JAD‐160017		0.18	10.1038/mp.2014.32		28.96	10.1038/mp.2014.32
					55	10.1038/mp.2014.32		21.21	10.1002/ana.22089		0.09	10.1056/NEJMra0909142		1.76	10.3233/JAD‐180121
					45	10.1016/j. brs.2014.11.020		20.78	10.1038/mp.2014.32		0.05	10.3233/JAD‐180121		1.65	10.1056/NEJMra0909142
					43	10.1001/jamaneurol.2017.3762		18.90	10.1016/j. brs.2014.11.020		0.05	10.3109/15622975.2010.538083		1.53	10.1016/j. jalz.2011.03.005
					41	10.1002/ana.22089		14.11	10.1016/j. jalz.2011.03.005		0.04	10.3233/JAD‐121579		1.46	10.3233/JAD‐121579
Overall DCA analysis					106	10.1016/j. neurobiolaging.2019.08.020		30.04	10.1016/j. neurobiolaging.2019.08.020		0.18	10.1038/mp.2014.32		28.96	10.1038/mp.2014.32
					102	10.1016/j. clinph.2019.11.002		29.49	10.1016/j. clinph.2015.02.001		0.15	10.1056/NEJMoa060281		13.46	10.1007/s00702‐012‐0902‐z
					96	10.1016/j. neuroimage.2017.12.048		27.39	10.1016/j. clinph.2019.11.002		0.15	10.1016/j. psychres.2011.06.006		6.91	10.1056/NEJMoa060281
					73	10.3233/JAD‐160017		26.40	10.3233/JAD‐160017		0.14	10.1007/s00702‐012‐0902‐z		5.08	10.1136/jnnp.2009.197848
					70	10.1016/j. clinph.2015.02.001		26.09	10.1016/j. neurobiolaging.2015.04.016		0.14	10.1056/NEJMoa0907083		4.43	10.1016/j. psychres.2011.06.006

Abbreviations: B, burst; CS, cluster size; CC, citation count; SMV, silhouette metric value; σ, centrality; ∑, sigma.

Cluster dependencies help researchers identify the interrelatedness and interdependencies between clusters of research domain within a specific area. By analyzing these dependencies, researchers can gain insights into the dynamics of the research landscape, including emerging trends, interconnections, and the diffusion of knowledge. As shown in Figure 5, the cluster dependencies were quite complex in this domain. Almost all the clusters have some dependent cluster, which shows that all clusters have nearly very tight interconnection in terms of their subfields or research themes. This representation highlights the interconnectedness and dependencies among clusters and their research themes.

In the third approach, cluster transformation over time (year) refers to the examination of how clusters of related research topic or themes evolve and change over different time period and provides valuable insights into the dynamics of research trends and the evolution of scientific knowledge. The analysis of cluster transformation unveils discernible patterns in research focus over time. Initially, in 2000, cluster #6, focusing on cognitive outcome, took center stage, suggesting foundational exploration within the field. By 2005, clusters #3 (nonmotor manifestation) and #5 (psychotic symptom) gained prominence, indicating a broadening scope of investigation. However, by 2010, the landscape shifted significantly, with clusters #3 (nonmotor manifestation), #9 (cognition‐focused intervention), #15 (anterior temporal lobe), #17 (normal aging), and #18 (REM sleep behavior disorder) emerging as pivotal areas of study. This period marked a transition towards more specialized research areas within the cognitive stimulation domain. In 2015, attention turned towards clusters #1 (functional evaluation), #2 (pathological ageing), #4 (nucleus basalis), #9 (cognition‐focused intervention), #11 (long‐term outcome), and #15 (anterior temporal lobe), indicating a focus on disease‐specific aspects, intervention strategies, and diagnostic methodologies were incorporated with cognitive stimulation. By 2020, clusters #0 (Parkinson's disease), #2 (pathological ageing), #4 (nucleus basalis), #7 (gamma frequency), #8 (nonmotor symptom), #11 (long‐term outcome), #12 (global cognition), #13 (cognition quality), #14 (systematic review), and #16 (disentangling gut‐brain connection) gained prominence, reflecting a continued diversification of research interests across various dimensions of the cognitive stimulation field. Following the COVID‐19 pandemic, in 2023, there was a notable concentration of studies within related clusters #0 (Parkinson's disease), #4 (nucleus basalis), #7 (gamma frequency), #12 (global cognition), #13 (cognition quality), and #14 (systematic review), suggesting a shift towards topics directly relevant to the pandemic's impact on neurological research. The same trend has continued into 2024. As shown in Figures 6 and 7, the thickness of connections between clusters serves as an indicator of the volume of studies conducted in a particular year, emphasizing the evolving research landscape and the responsiveness of scholarly inquiry to emerging trends and challenges.

## 4 DISCUSSION

Over the span of 36 years from 1989 to 2024, the WoSCC database witnessed a significant surge in published and indexed documents, totaling 4156. A particularly noteworthy increase in publications observed between 2019 and 2023, which can be attributed to heightened attention and recognition within the scientific community, notably post the COVID‐19 pandemic. The year 2022 marked the highest publications with 441 articles, the highest number since 1989, showcasing a dynamic landscape of cognitive stimulation research. This section further discusses the key findings and trends aligned with the proposed aims of this study.

### 4.1 Evolution of research over three decades and influential researchers

Research activities evolved substantially over the past three decades. A notable acceleration in research output and innovation occurred after 2010, driven by advancements in technology, interdisciplinary approaches, and heightened global attention to neurodegenerative diseases. This study further aimed to identify the most influential authors in this study field. Based on citation counts, *Folstein, MF*, is renowned for the development of the mini‐mental state examination (MMSE), a key tool in cognitive assessment.^67^ *Petersen, RC* contributed to defining MCI as a critical clinical entity in the study of cognitive decline.^68^ Further, *Nitsche, MA* focused on noninvasive brain stimulation techniques, such as tDCS, in enhancing cognitive functions.^69^ *Cotelli, M* explored the efficacy of cognitive stimulation in Alzheimer's patients, advancing therapeutic approaches for cognitive impairments.^70^ These authors significantly influenced the landscape of cognitive stimulation research through their pioneering work in diagnostic tools, treatment techniques, and theoretical advancements.

On the basis of centrality in ACA, *Aaslid, R* made significant advancements in brain imaging and cerebral blood flow measurement techniques, which are vital in understanding brain function during cognitive tasks.^71^ *Dubois, B* is recognized for his work in AD, contributing to diagnostic frameworks that help identify early cognitive impairments.^72^ *Barker, AT* pioneered TMS, a technique used to stimulate brain areas involved in cognition.^73^ *Benabid, AL* is known for his groundbreaking work in DBS, which has therapeutic applications in cognitive and motor disorders.^13^ These authors' high centrality values highlight their crucial roles in developing methodologies and treatments that have advanced the field of cognitive stimulation and rehabilitation.

Based on burst analysis in ACA, the following five authors have shown significant short‐term research impact in cognitive stimulation studies for cognitive impairments. *Chou, YH* has contributed to the recent surge in noninvasive brain stimulation research, particularly in applying tDCS to enhance cognitive functions in aging and neurodegenerative diseases.^29^ *McKhann, G* is notable for his work on the diagnostic criteria for AD, which remains a foundation for clinical research and diagnosis.^74^ *Krack, P* explored DBS as a treatment for PD, with implications for cognitive improvements in related disorders.^75^ *Fahn, S* made influential contributions in movement disorders and neurodegenerative diseases, connecting motor dysfunction with cognitive decline.^76^ *Alagona, G* investigated motor cortex excitability and its relation to cognitive performance, advancing the understanding of brain stimulation's role in cognitive rehabilitation.^77^ These high burst values highlight their pivotal contributions during key periods of research innovation.

### 4.2 Geographical contributions and prominent institutions

In analyzing the most influential countries or regions, the United States emerged as a dominant force, leading in citation counts, centrality, and sigma value. Notable contributors like Italy, China, and Germany showcased the diverse global contributions to this brain stimulation domain. Meanwhile, in the realm of influential institutions, the University of London and the University of Manchester stood out for their citation counts and sudden increases in citation frequency, whereas the APHP led in centrality value, and Capital Medical University in China took the lead in sigma value.

### 4.3 Interdisciplinary connections

Dual‐map overlay analysis revealed strong interdisciplinary connections, with cognitive neuroscience, neurology, and rehabilitation science as primary contributions, and these connections underscore the multifaceted nature of brain stimulation research, integrating insights from diverse fields to address complex cognitive impairments. Specifically, the analysis highlights that research outputs in brain stimulation bridge the gap between basic neuroscience and applied clinical practices. The convergence of these fields emphasizes the critical role of interdisciplinary collaboration in advancing brain stimulation research. This integration enables researchers to develop innovative techniques, such as noninvasive brain stimulation, which can improve cognitive functions and rehabilitate patients with neurodegenerative disorders. The findings of this analysis conclude that brain stimulation research not only spans multiple disciplines but also benefits from their combined theoretical and practical contributions, fostering a comprehensive approach to understand and addressing cognitive impairments.

### 4.4 Emerging research clusters and global trends on brain stimulation studies

DCA analysis illuminated clusters of related research topics, demonstrating distinct patterns in research focus over time and reflecting the evolving landscape of scientific inquiry within the cognitive stimulation domain. These comprehensive analyses collectively offer insights into the multifaceted aspects of cognitive stimulation research, its global impact, interdisciplinary nature, and evolving trends over time. The top five clusters, based on cluster size, are discussed further to understand the emerging trends of this study domain.

#### 4.4.1 Cluster #0: Parkinson's disease

Cluster #0 represents the largest cluster in the DCA analysis, comprising 162 documents/articles with a high Silhouette value of 0.902, indicating a well‐defined and coherent grouping. This cluster focuses on PD and its related neurodegenerative aspects, particularly emphasizing the diagnostic and therapeutic applications of noninvasive brain stimulation, such as TMS. Several key studies explore the potential of brain stimulation as a diagnostic tool and therapeutic intervention in neurodegenerative conditions. For instance, a study highlights the contributions of TMS to diagnosing and treating dementia, a major comorbidity in PD, suggesting its role in early diagnosis and treatment of cognitive impairments in patients with PD.^78^ Similarly, another study provides a comprehensive systematic review that examines the effectiveness of brain stimulation in enhancing cognitive function, particularly in PD and movement disorders, illustrating its potential to improve cognitive function.^79^ These studies underscore a growing interest in using TMS to address not only as a treatment for motor symptoms but also cognitive impairments in PD.^4^, ^78^ Moreover, a novel perspective advocates for “Brain Stimulation 2.0,” a personalized approach to brain stimulation based on individual neurocognitive profiles, which could be crucial for managing PD given its heterogeneity in motor and cognitive symptoms.^30^ Other highly cited authors in this cluster contribute to understanding how TMS can modulate cortical excitability to alleviate both motor and cognitive symptoms in PD.^29^, ^79^ Additional research highlights the use of TMS to improve connectivity between cortical and subcortical regions in PD.^66^ In summary, the research in this cluster substantially advances our understanding of neurodegenerative pathways contributing to cognitive decline and the therapeutic potential of brain stimulation for enhancing brain plasticity. As the field moves toward more personalized and integrative treatments, these studies provide a solid foundation for future research, particularly emphasizing the need to address cognitive symptoms as integral to PD management.

#### 4.4.2 Cluster #1: Functional evaluation

Cluster #1 is the second largest cluster identified in the analysis, consisting of 121 documents/articles with a Silhouette value of 0.936, reflecting a distinct and well‐defined focus on the functional evaluation of cognitive decline and noninvasive brain stimulation techniques, particularly in the context of AD, healthy aging, and other neurodegenerative conditions. Influential studies within this cluster provide a systematic review on the therapeutic potential of noninvasive brain stimulation in AD, laying the groundwork for future interventions.^80^ Expanding on this, a comprehensive meta‐analysis on the effects of brain stimulation on cognitive function in both healthy aging individuals and those with AD further extends this line of inquiry.^81^ Another study offers foundational insights into the clinical neurophysiology of the aging brain, tracing the trajectory from normal aging to neurodegeneration, which has been pivotal in framing how brain stimulation can intervene in the early stages of cognitive decline.^82^ Further research emphasizes the role of neurostimulation not only as a therapeutic tool but also as a means of functional evaluation in AD.^83^ This multifaceted approach highlights the potential of brain stimulation in assessing the progression of cognitive decline and the efficacy of interventions, providing a noninvasive method for clinicians to track treatment outcomes.

#### 4.4.3 Cluster #2: Pathological aging

Cluster #2, the third‐largest in the DCA analysis, comprises 113 documents/articles with a Silhouette value of 0.915. This cluster mainly focuses on the pathological aspects of aging, particularly in relation to cognitive decline and neurodegenerative diseases like AD. The research within this cluster emphasizes the use of tDCS, to address the cognitive impairments associated with pathological aging.

Several influential articles have significantly shaped the understanding of pathological aging and the potential therapeutic applications of tDCS. One article presents evidence‐based guidelines on the therapeutic use of tDCS, offering a comprehensive review of the current state of knowledge and recommendations for its clinical application in treating neurodegenerative diseases, including AD.^34^, ^79^, ^84^ These guidelines are crucial in guiding both research and clinical practice, ensuring that the application of tDCS is grounded in scientific evidence. Further exploration reveals how brain stimulation techniques, especially tDCS, can mitigate the cognitive deficits in AD by targeting underlying neural mechanisms.^85^ For instance, a study demonstrated that anodal tDCS applied to the left dorsolateral prefrontal cortex improved performance in a verbal fluency task among AD patients.^86^ The neural mechanism underlying this effect involves the modulation of cortical excitability and synaptic plasticity, facilitated by changes in long‐term potentiation processes in the stimulated area, which is crucial for memory and learning. The potentials and limitations of tDCS are further examined, emphasizing that while the technique shows promise, its success depends on various factors, such as the specific cognitive domain targeted and individual patient differences.^87^

Further research demonstrates the potential of tDCS to enhance memory function in individuals with MCI, often one of the earliest signs of pathological aging.^88^ Additionally, a study explores the combined effects of exercise and tDCS, hypothesizing that this combination could enhance cognitive functions in MCI and AD populations more effectively than either intervention alone.^89^

#### 4.4.4 Cluster #3: Nonmotor manifestation

Cluster #3, the fourth largest cluster, comprises 108 members and has a Silhouette value of 0.954. This cluster reflecting a concentrated focus on the nonmotor manifestations of PD. While PD is traditionally recognized for its motor symptoms, this cluster emphasizes the equally significant nonmotor symptoms that severely impact patients' quality of life, such as cognitive decline, mood disorders, and autonomic dysfunction.

A seminal review presents a thorough examination of PD treatment, covering both motor and nonmotor symptoms, lays out therapeutic strategies aimed at holistic patient care.^90^ This study serves as a cornerstone in PD research, offering a detailed review of the therapeutic strategies available for managing the full spectrum of PD symptoms. The extensive coverage of nonmotor symptoms in this article highlights the importance of addressing these aspects to improve overall patient care. Another critical study explores the challenge of managing levodopa‐unresponsive symptoms, emphasizing the need for innovative treatments that go beyond conventional motor symptom management.^91^ This study underscores the complexity of PD and the necessity for a holistic approach to treatment that addresses both motor and nonmotor symptoms. Further study shifts the focus toward the neuropsychiatric dimensions of PD, underscoring the profound impact of cognitive and mood disorders.^92^ This perspective reframes PD as the quintessential neuropsychiatric disorder, suggesting that these nonmotor manifestations are not secondary but rather integral components of the disease process. This perspective is crucial for developing more comprehensive treatment strategies that include psychiatric and cognitive interventions alongside traditional motor symptom management.

#### 4.4.5 Cluster #4: Nucleus basalis

Cluster #4, the fifth largest cluster in the DCA analysis, comprises 102 members with a Silhouette value of 0.962, indicating a very cohesive and focused body of research. This cluster centers on the role of the nucleus basalis, a brain region critical for cognitive functions, particularly in the context of neurodegenerative diseases like AD and PD. This cluster explores neuromodulation techniques for treating AD, particularly discusses both current and experimental approaches to modulating the nucleus basalis to slow cognitive decline.^93^ The emphasis on neuromodulation reflects a growing interest in developing noninvasive treatments that can potentially alter the course of neurodegenerative diseases. Another study examines the use of DBS in the memory circuit, particularly its application in improving cognitive function in AD.^94^ This study underscores the potential of DBS to enhance cognitive functions by directly targeting the nucleus basalis, offering hope for new therapeutic strategies in the management of dementia‐related disorders. Additional research explores DBS in the context of dementia‐related disorders, emphasizing its role in modifying disease progression and tried to study how DBS to slow or even halt the progression of cognitive decline by targeting key brain regions like the nucleus basalis.^95^ These studies collectively underscore the therapeutic potential of DBS and suggest that it may go beyond managing symptoms to directly address the underlying pathology of neurodegenerative conditions.

#### 4.4.6 Integrated view of research hotspots and trends

The clustering analysis of brain stimulation research reveals distinct yet interconnected themes across the top five clusters. Each cluster focuses on different neurodegenerative conditions and therapeutic interventions, with potential correlations emerging between them based on shared techniques, clinical implications, and the integration of brain stimulation for cognitive and motor rehabilitation, particularly PD and AD. Emerging trends include the move toward personalized brain stimulation (Cluster #0), combining neurostimulation with other interventions like exercise (Cluster #2), and the application of DBS to target specific neural circuits (Cluster #4) for cognitive enhancement. A critical analysis of these clusters shows a growing integration of therapeutic and evaluative approaches, with an increasing emphasis on understanding how nonmotor symptoms (Cluster #3) and pathological aging (Cluster #2) can be mitigated through both noninvasive and invasive neurostimulation techniques.

#### 4.4.7 Other emerging clusters from DCA analysis

In addition to the top‐5 clusters, it is important to point out other few clusters which are emerged recently. Specifically, cluster #7 labeled as gamma frequency by LLR and mean year as 2019, followed by cluster #12 (global cognition; mean year‐2019), cluster #25 (curcumin analog tml‐6; mean year‐2019), and cluster #13 (cognition quality; mean year‐2018). Within these clusters, particularly emphasis on various novel brain stimulation techniques such as fast gamma magnetic stimulation (FGMS),^96^ a combination of training and Aβ‐immunotherapy for AD treatment,^97^ paired‐associative stimulation with TMS (PAS‐TMS), and TBS,^98^ which represent the emerging trends in recently established clusters.

### 4.5 Study implications and directions for future research

Based on various analyses and the growing number of publications in this brain stimulation research domain, this scientometric review study found several significant implications, research gaps, and directions for future research in this domain. The implications drawn from the analyses underscore the diverse applications of brain stimulation techniques in cognitive enhancement, symptom management, and understanding the neural mechanisms underlying neurodegenerative and psychiatric disorders. The identified research gap and future directions include:

– Noninvasive brain stimulation techniques, such as tDCS and rTMS, exhibit great potential for improving cognitive function in AD and MCI patients. Future research could be more comprehensive into optimizing stimulation protocols, targeting specific brain regions, and exploring long‐term effects and mechanisms underlying cognitive enhancement.
– There is a research scope on refining DBS protocols, identifying patient subgroups that may benefit most, and elucidating the neural mechanisms underlying DBS effects in AD.
– Longitudinal studies provide insights into the sustained effects and progression over time following brain stimulation interventions. Future research should focus on long‐term monitoring of patients undergoing brain stimulation, identifying influencing treatment outcomes, and develop individualized intervention strategies.

In summary, future research should aim to refine existing protocols, explore novel techniques, and elucidate the long‐term effects and underlying mechanisms to advance the field of brain stimulation in clinical practice.

### 4.6 Limitations of this study

This study is limited to the WoSCC database, an exceptional and widely recognized international database as a primary source of almost all bibliometric research, but does not cover the remaining databases such as Scopus or PUBMED. As a result, important studies published in journals indexed elsewhere may be overlooked, leading to incomplete data representation. In another, this study data set is limited with English‐language publications only, which may lead to geographical and cultural biases. Despite these limitations, the author believes that this study could serve as a dynamic first‐in‐kind of bibliometric review within the brain stimulation domain and could be a useful resource for future researchers and practitioners in this field.

## 5 CONCLUSION

This study explores the emerging field of noninvasive brain stimulation techniques, particularly their applications in addressing cognitive impairment and related disorders. Through extensive bibliometric analyses from 1989 to 2024, significant trends have emerged, indicating a notable increase in scholarly output, particularly in recent years (2019–2023). The United States, alongside institutions like the University of London and the University of Manchester, emerged as key contributors to this study domain. DCA analysis revealed distinct clusters of research, with TMS and rTMS prominently featured. Additionally, techniques such as FGMS, PAS‐TMS, and TBS, were identified as emerging brain stimulation techniques recently, and these valuable insights highlight the importance of continued exploration to advance our understanding and noninvasive therapeutic interventions for cognitive impairments.

## AUTHOR CONTRIBUTIONS

Mani Abdul Karim contributed to the entire work, including the study design, developing the search criteria, conducting analyses using CiteSpace, and writing the entire manuscript.

## CONFLICT OF INTEREST STATEMENT

The author declares that the research was conducted in the absence of any commercial or financial relationships that could be construed as a potential conflict of interest.

## ETHICS STATEMENT

This study involves no primary human participants, relying solely on secondary data analysis from publicly available bibliometric records. As such, ethical approval is not required. Data will be handled with transparency, following established academic guidelines for bibliometric research.

## Supporting information

Supporting information.

## Data Availability

The data that support the findings of this study is available in a public repository at psyarxiv (DOI: 10.17605/OSF.IO/XS4HJ).
