# Supplementary material for: Mapping the landscape of brain stimulation research: A global scientometric review on cognitive impairment
Source: Ibrain. 2025 Mar 22;11(2):185–204. doi: 10.1002/ibra.12194 (PMC12177676; doi:10.1002/ibra.12194)
Supplement: Supplementary file 1 — Supporting information. [file IBRA-11-185-s001.docx]

**Supplementary figure**


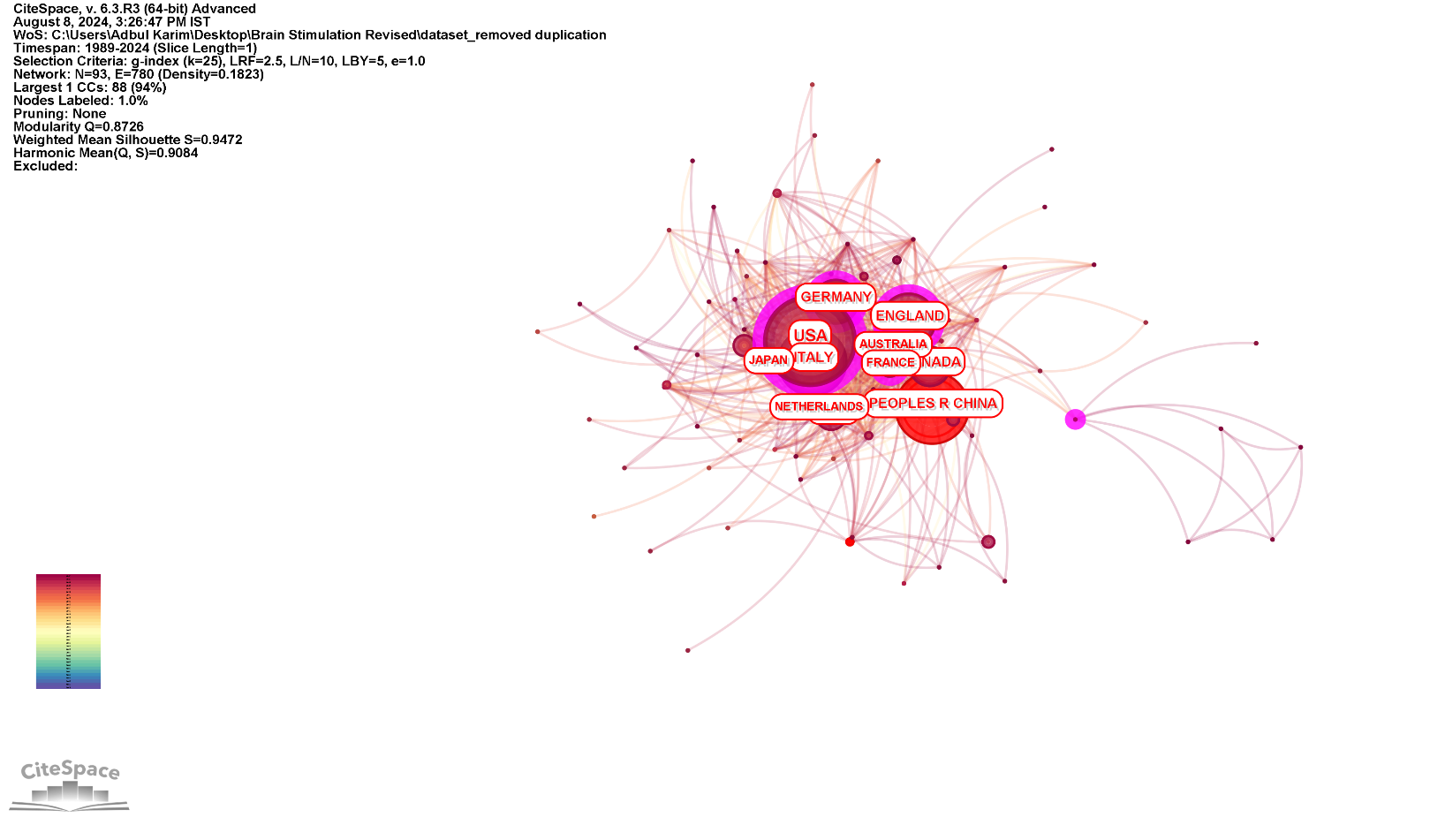


***Supplementary figure 1.*** Country network analysis using CiteSpace

*Note:* Pink ring denotes to major contribution of the country


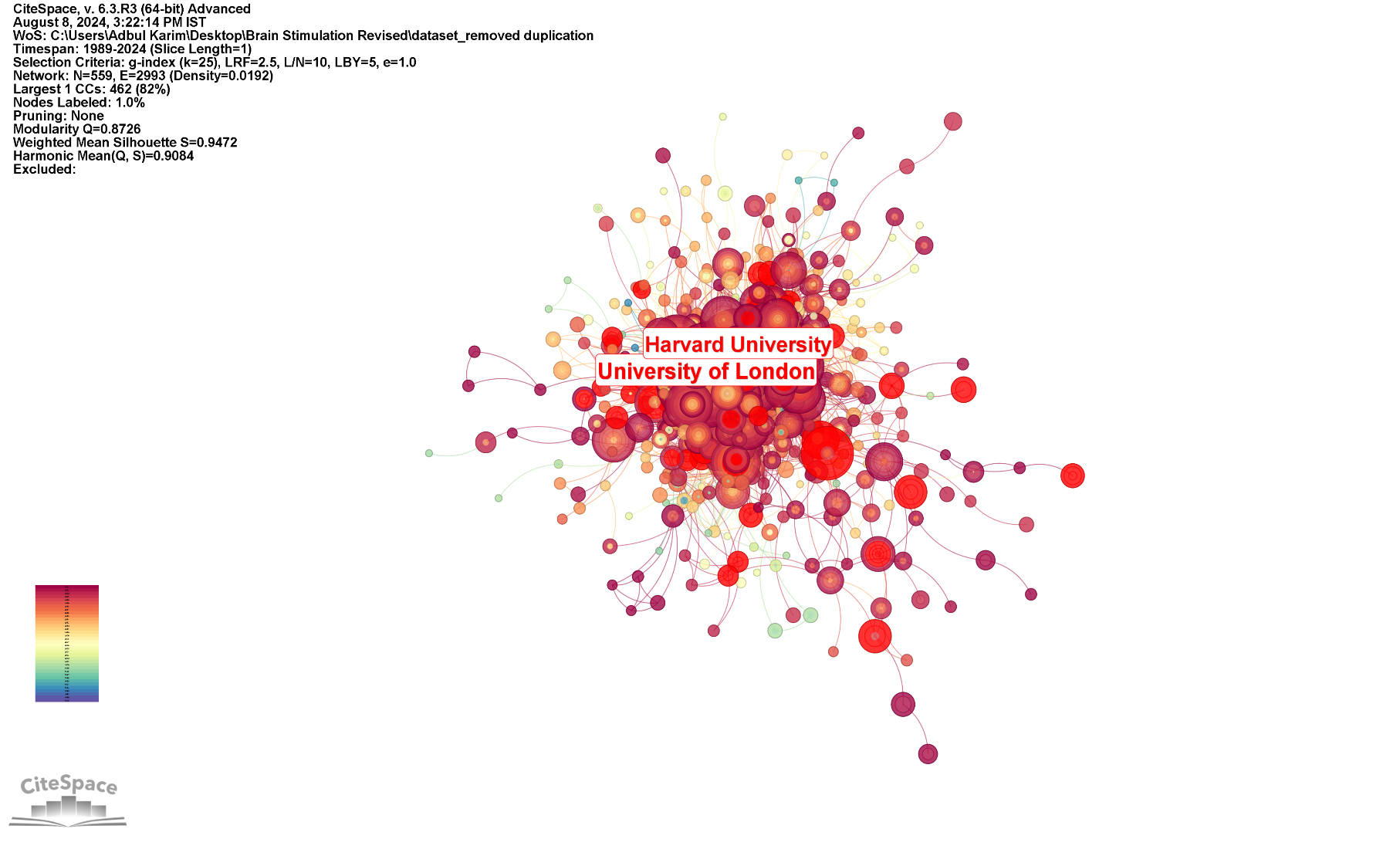


***Supplementary figure 2.*** Institutional network analysis using CiteSpace

*Note:* The size of the ring denotes to major contribution of the institution


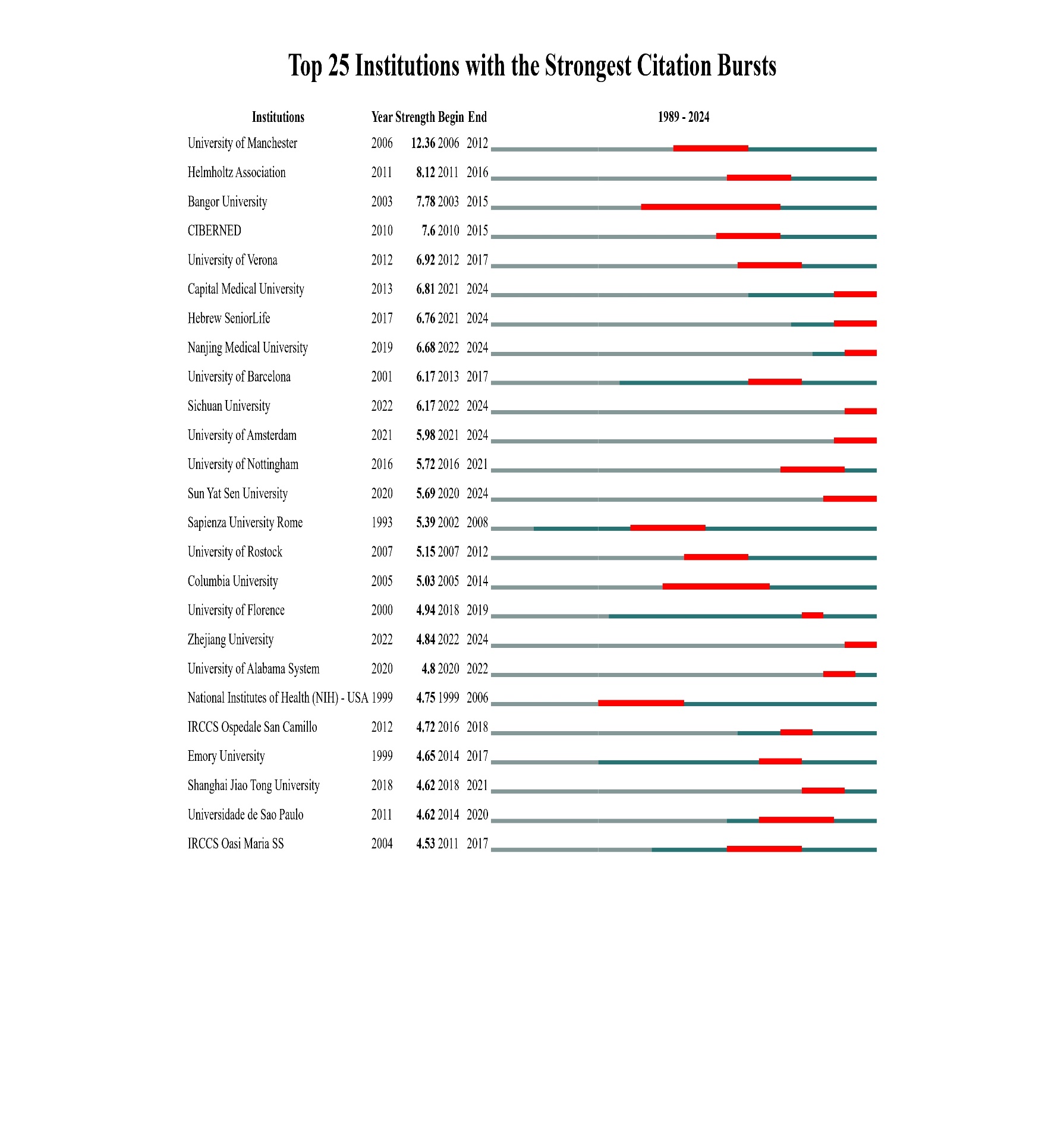


***Supplementary figure 3.*** Top-25 Institutions with the Strongest Citation Bursts based on Bibliometric Analysis using CiteSpace on cognitive stimulation studies for cognitive impairment and related disorders


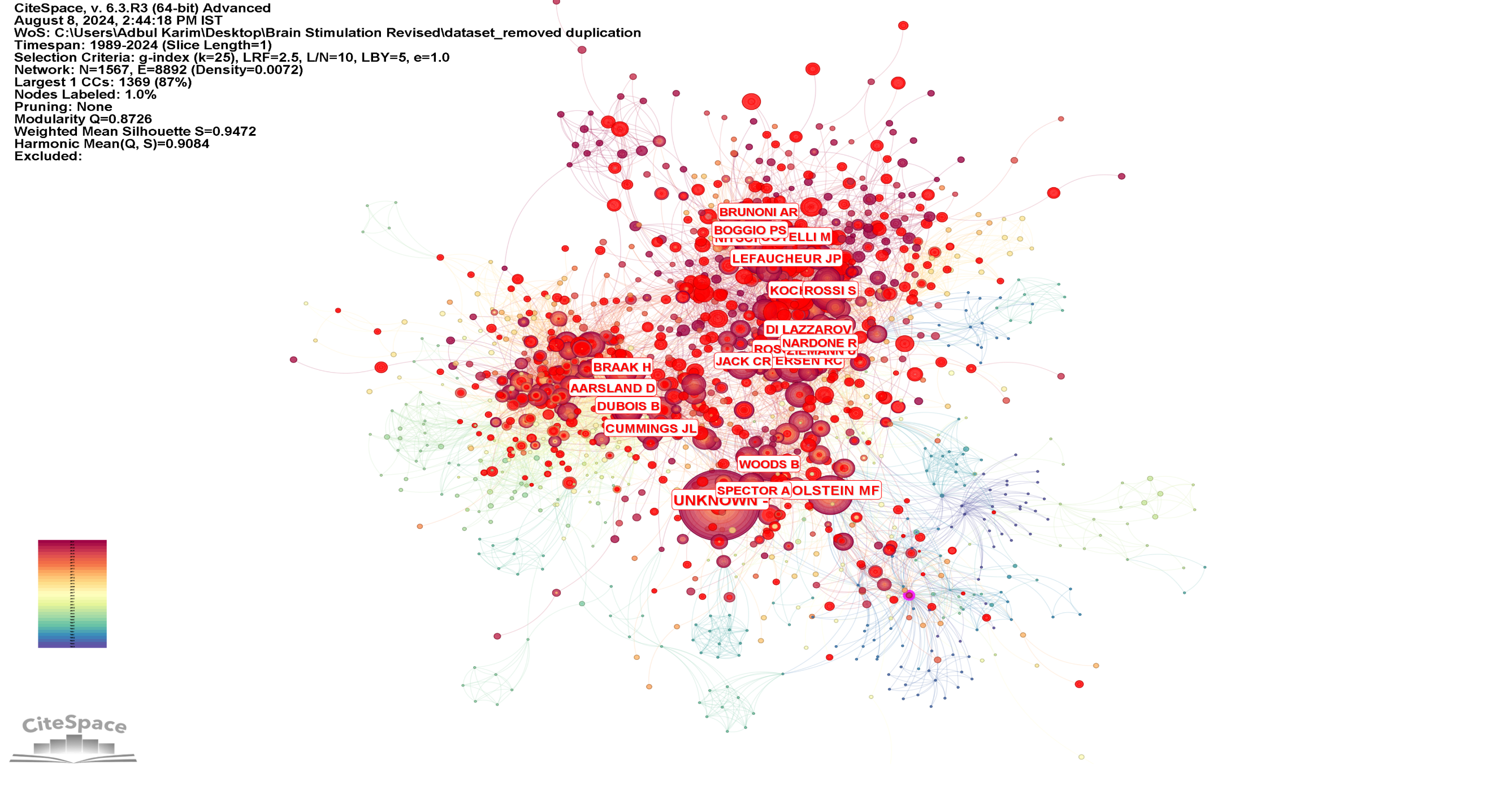


***Supplementary figure 4.*** The landscape mapping view of author co-citation analysis (ACA) of brain stimulation studies for cognitive impairment and related disorders, generated by top 50 per slice between 1989-2024.


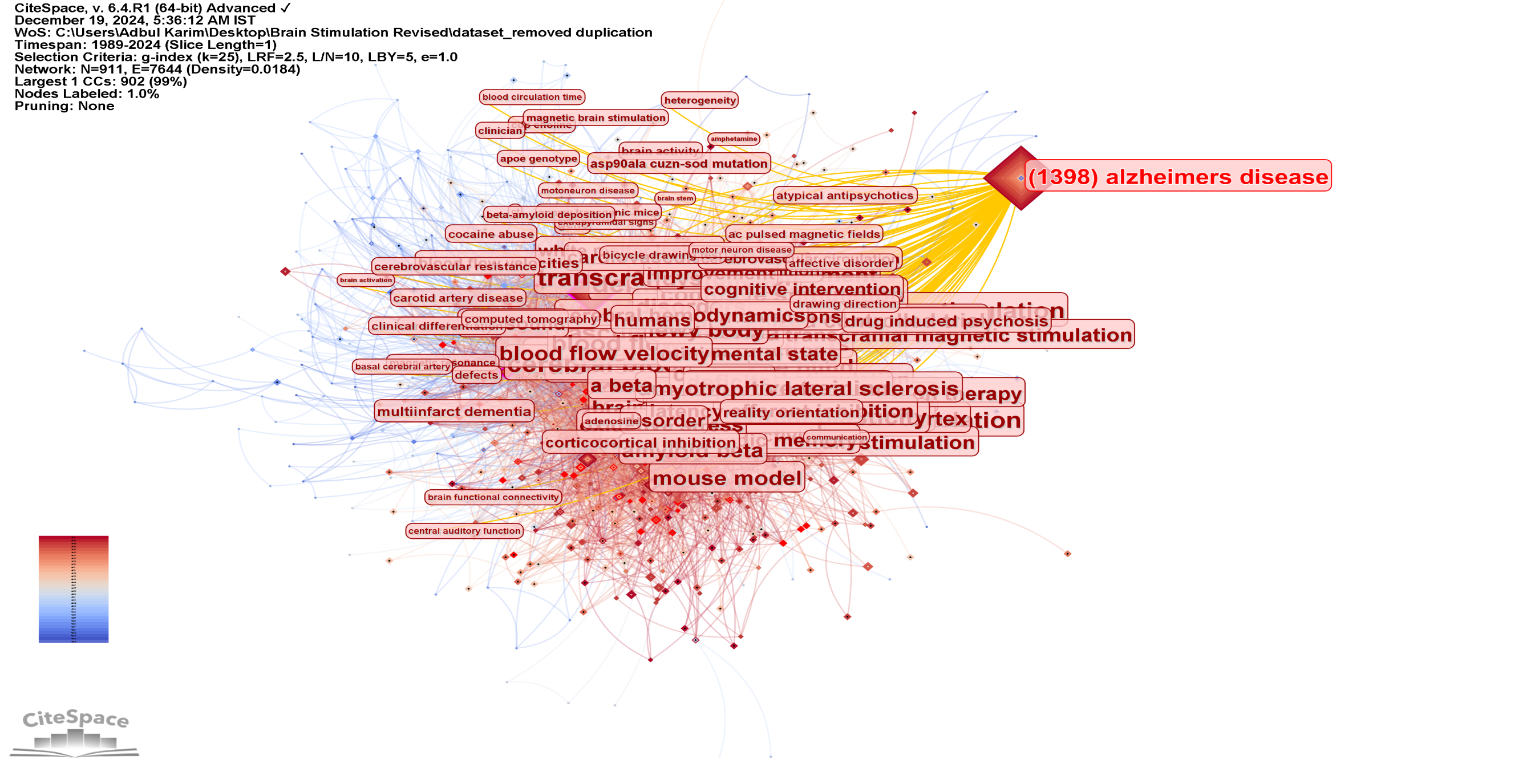


***Supplementary figure 5.*** Keyword co-occurrence using CiteSpace

**Supplementary table**

**Supplementary table 1.**

Top-10 countries in cognitive stimulation research from 1989 to 2024 based on citation counts, burst, centrality, and sigma value

| **Rank** | **Citation Counts** | **Country** | **Burst** | **Country** | **Centrality** | **Country** | **Sigma** | **Country** |
| --- | --- | --- | --- | --- | --- | --- | --- | --- |
| 1 | 1117 | USA | 72.31 | China | 0.37 | USA | 3.68 | USA |
| 2 | 626 | China | 9.08 | Wales | 0.20 | Egypt | 2.16 | Germany |
| 3 | 598 | Italy | 7.78 | Germany | 0.16 | France | 1.79 | China |
| 4 | 524 | England | 6.56 | Austria | 0.13 | England | 1.78 | France |
| 5 | 377 | Germany | 4.25 | Scotland | 0.10 | Germany | 1.34 | England |
| 6 | 318 | Canada | 4.15 | USA | 0.10 | Australia | 1.32 | Japan |
| 7 | 253 | Spain | 3.94 | Japan | 0.10 | Canada | 1.18 | Canada |
| 8 | 233 | Australia | 3.87 | France | 0.08 | Brazil | 1.13 | Scotland |
| 9 | 199 | France | 3.33 | Belgium | 0.07 | Japan | 1.11 | Netherlands |
| 10 | 146 | Netherlands | 3.19 | Hungary | 0.06 | Spain | 1.11 | Austria |

*Source:* The data were derived from analysis (CiteSpace) using dataset retrieved from Web of Science core collection (WoSCC)

**Supplementary table 2.**

Top-10 academic institutions published records during the period 1989-2024 on cognitive stimulation related studies for cognitive impairment and related disorders

| **Rank** | **CC** | **Institution** | **Centrality** | **Institution** | **Sigma** | **Institution** |
| --- | --- | --- | --- | --- | --- | --- |
| 1 | 235 | University of London | 0.10 | APHP | 1.77 | Capital Medical University |
| 2 | 183 | Harvard University | 0.09 | Capital Medical University | 1.49 | APHP |
| 3 | 180 | University College London | 0.06 | INSERM | 1.36 | University of Manchester |
| 4 | 171 | University of Toronto | 0.06 | University of Pennsylvania | 1.22 | University of Pennsylvania |
| 5 | 139 | Harvard Medical School | 0.06 | Johns Hopkins University | 1.22 | University of Barcelona |
| 6 | 114 | INSERM | 0.06 | University of California System | 1.17 | National Institutes of Health |
| 7 | 112 | University of California System | 0.05 | University Health Network Toronto | 1.16 | Sapienza University Rome |
| 8 | 95 | University Health Network Toronto | 0.05 | Beth Israel Deaconess Medical Center | 1.15 | Shanghai Jiao Tong University |
| 9 | 78 | US Department of Veterans Affairs | 0.05 | US Department of Veterans Affairs | 1.15 | Bangor University |
| 10 | 78 | Veterans Health Administration (VHA) | 0.05 | Newcastle University | 1.12 | Columbia University |

*Source:* The data were derived from analysis (CiteSpace) using dataset retrieved from Web of Science Core Collection (WoSCC).

Abbreviation: *CC, Citation Counts; INSERM, Institut National de la Sante et de la Recherche Medicale; APHP, Assistance Publique Hopitaux Paris.*

**Supplementary table 3.**

*The top-5 authors during the period of 1989-2024 on cognitive stimulation related studies for cognitive impairment and related disorders, listed with publication records, citation counts (CC), centrality and burst*

| **Rank** | **Publication records** | **Author** | **CC** | **Cited Author** | **σ** | **Cited Author** | **Burst** | **Cited Author** |
| --- | --- | --- | --- | --- | --- | --- | --- | --- |
| 1 | 60 | Spector, A | 557 | Folstein, MF, 1989 | 0.15 | Aaslid, R, 1989 | 32.80 | Chou, YH, 2020 |
| 2 | 48 | Orrell, M | 315 | Petersen, RC, 2007 | 0.09 | Dubois, B, 1999 | 28.09 | McKhann, G, 1989 |
| 3 | 46 | Lozano, AM | 312 | Nitche, MA, 2011 | 0.08 | Barker, AT, 1992 | 22.64 | Fahn, S, 2000 |
| 4 | 45 | Pascual-leone, A | 284 | DI Lazzarov, 2004 | 0.07 | Benabid, AL, 1998 | 22.26 | Krack, P, 2000 |
| 5 | 44 | Koch, G | 282 | Cotelli, M, 2008 | 0.06 | Mckeith, IG, 1999 | 21.01 | Alagona, G, 2004 |

Abbreviation: CC, Citation Count; **σ**, Centrality

**Supplementary table 4.**

Top-10 keywords related to cognitive stimulation related studies for cognitive impairment and related disorders, listed based on citation counts (CC), centrality, and burst

| **Rank** | **CC** | **Keyword** | **σ** | **Keywords** | **Burst** | **Keyword** |
| --- | --- | --- | --- | --- | --- | --- |
| 1 | 1398 | alzheimers disease | 0.11 | dementia | 26.51 | transcranial doppler |
| 2 | 833 | dementia | 0.10 | cerebral blood flow | 14.99 | subthalamic nucleus stimulation |
| 3 | 819 | transcranial magnetic stimulation | 0.09 | alzheimers disease | 14.11 | cerebral blood flow |
| 4 | 789 | deep brain stimulation | 0.09 | brain | 14.00 | vascular dementia |
| 5 | 678 | parkinsons disease | 0.06 | transcranial doppler | 13.45 | cardiopulmonary bypass |
| 6 | 629 | mild cognitive impairment | 0.06 | deep brain stimulation | 11.38 | transcranial doppler sonography |
| 7 | 531 | cognitive impairment | 0.06 | parkinsons disease | 11.11 | follow up |
| 8 | 340 | impairment | 0.05 | blood flow | 10.00 | blood flow |
| 9 | 300 | memory | 0.05 | disease | 9.59 | parkinsons disease |
| 10 | 279 | older adults | 0.04 | basal ganglia | 9.44 | white matter lesions |

*Source:* The data were derived from analysis (CiteSpace) of keyword analysis using dataset retrieved from Web of Science Core Collection (WoSCC)

Abbreviation: *CC, Citation Counts;* σ, centrality
